# First report of Q fever in Oman.

**DOI:** 10.3201/eid0601.000114

**Published:** 2000

**Authors:** E M Scrimgeour, W J Johnston, S H Al Dhahry, H S El-Khatim, V John, M Musa

**Affiliations:** Sultan Qaboos University, Al-Khod, Sultanate of Oman. scrim@squ.edu.om

## Abstract

Although serologic evidence suggests the presence of Q fever in humans and animals in Saudi Arabia and the United Arab Emirates, acute Q fever has not been reported on the Arabian Peninsula. We report the first two cases of acute Q fever in Oman.


### First Report of Q Fever in Oman

**Euan M. Scrimgeour,* William J. Johnston,* Said H.S. Al Dhahry,***
**Hussein S. El-Khatim,† Valliath John,‡ and Mohamed Musa‡**
*Sultan Qaboos University, Al-Khod, Sultanate of Oman;
†Sultan Qaboos University Hospital, Al-Khod, Sultanate of Oman;
‡Royal Hospital, Muscat, Sultanate of Oman

Although serologic evidence suggests the presence of Q fever in humans and animals in Saudi Arabia and the United Arab Emirates, acute Q fever has not been reported on the Arabian Peninsula. We report the first two cases of acute Q fever in Oman.

Acute Q fever has not been reported on the Arabian Peninsula; however, serologic studies have documented *Coxiella burnetii* in eastern Saudi Arabia. In Riyadh, in the late 1960s, 8.4% of residents >5 years of age were seropositive (1). Veterinary studies in Saudi Arabia have shown that Q fever occurs in livestock (2) and in Arabian oryx (*Oryx leucoryx*) at the National Wildlife Research Centre at Taif (3). In Abu Dhabi, United Arab Emirates (UAE), the disease was reported in racing camels (4).

Acute Q fever is usually a nonspecific febrile illness, often with atypical pneumonia or transient hepatitis. Seroconversion without symptoms is common, especially in children (5). As a rule, it is self-limiting and resolves without treatment, but some untreated cases may progress to chronic Q fever (endocarditis, granulomatous hepatitis, osteomyelitis, interstitial pulmonary fibrosis) (6). Pericarditis is rare (7), mostly reported from France (6,8) and Spain (9), and sporadically worldwide (10).

In acute Q fever, diagnosis is supported by a fourfold increase in antibody titers to *C. burnetii* in acute- and convalescent-phase serum samples by complement-fixation test (CFT) or indirect immunofluorescence assay (IF). The IF is more sensitive and specific than CFT in chronic Q fever. In the National Reference Center in France, the diagnosis of acute infection is supported by IgG phase II $\geq$ 1:200, IgM phase II $\geq$ 1:50; and of chronic disease, IgG phase I $\geq$ 1:800 (11). Diagnosis can also be made by identifying *C. burnetii* in tissue culture or its antigens by polymerase chain reaction (PCR) in biopsy specimens. We report the first two human cases of Q fever in Oman.

Address for correspondence: Euan M. Scrimgeour, Department of Medicine, Sultan Qaboos University, P.O. Box 35, Al-Khod, 123, Sultanate of Oman; fax: 968-513-419; e-mail: scrim@squ.edu.om.

### Case 1

A 45-year-old Omani woman was hospitalized on December 13, 1997, with a 4-week history of fever and productive cough. The patient's temperature was 39°C, she was jaundiced, and she had signs of right lower lobe pneumonia, which was confirmed by X-ray (Figure 1).

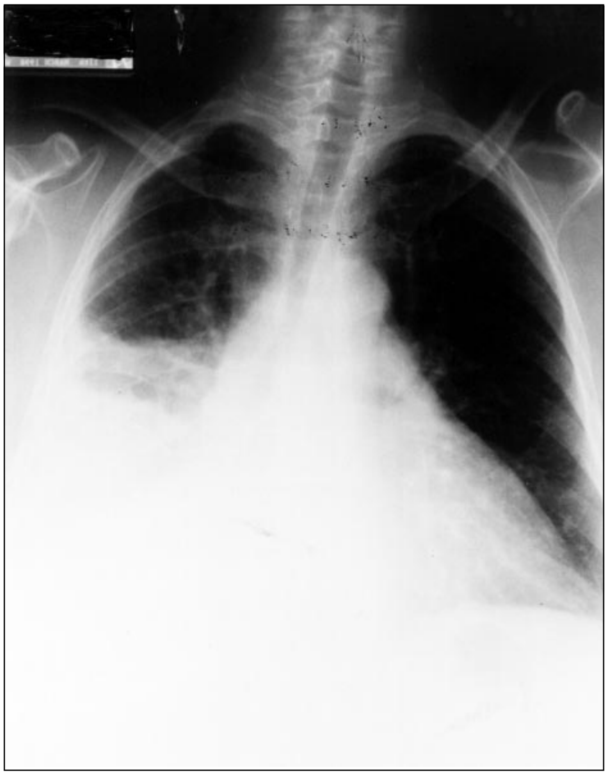

Figure 1. Q-fever pneumonia.

Hemoglobin was 9.5 g/dL, white blood cells 6.6 x $10^9$/L (neutrophils 76%, lymphocytes 14.2%), platelets 317 x $10^9$/L, and erythrocyte sedimentation rate 101 mm/hour. An electrocardiogram showed nonspecific abnormalities, but an echocardiogram revealed a small pericardial effusion. Liver function tests showed normal serum bilirubin, serum aspartate aminotransferase 111 μ/L (normal range 14-42 μ/L), serum alkaline phosphatase 256 μ/L (normal range 3-210 μ/L), and serum albumen 26 g/L. Community-acquired pneumonia, with pericarditis and hepatitis, was diagnosed, and the patient was treated with intravenous erythromycin, 500 mg 4 times a day.

Since the patient lived on a farm with cattle and goats, Q fever was considered, although *C. burnetii* infection had never been reported in Oman. Sera were sent to England for Q-fever antibody tests, which were not available locally. The patient became afebrile, signs of pneumonia resolved, and she was discharged on December 31, 1997 (before serologic results were received).

On January 12, 1998, the patient had signs of constrictive pericarditis and small bilateral pleural effusions. The results of CFT for Q fever were total antibody titers to phase-II antigen ≥ 1:80 (threshold ≥ 1:10). Phase-I antigen and immunoglobulin (Ig) M tests were not performed, but the results indicated recent Q-fever infection, so doxycycline (100 mg twice a day) was prescribed. Repeat serologic tests (January 12, 1998) revealed total CFT antibody titers to phase-I and phase-II IgG antigens ≥ 1:40 (threshold ≥ 1:10); and IgM and IgA < 1:10 (threshold ≥ 1:10). These results were interpreted as evidence that *C. burnetii* infection was the probable cause of chronic pericarditis.

Echocardiography revealed a grossly thickened pericardium with abnormal septal motion encroaching on the right ventricular cavity in diastole, and the CT scan confirmed a diffusely thickened pericardium (Figure 2). Cardiac catheterization revealed normal left ventricular systolic function, but the central venous pressure was 26 cm. On April 5, at pericardiectomy, the pericardium was 1 cm thick and densely adherent to the heart. Histopathologic examination revealed granulomatous pericarditis without evidence of tuberculosis (Figure 3). The patient recovered well after surgery.

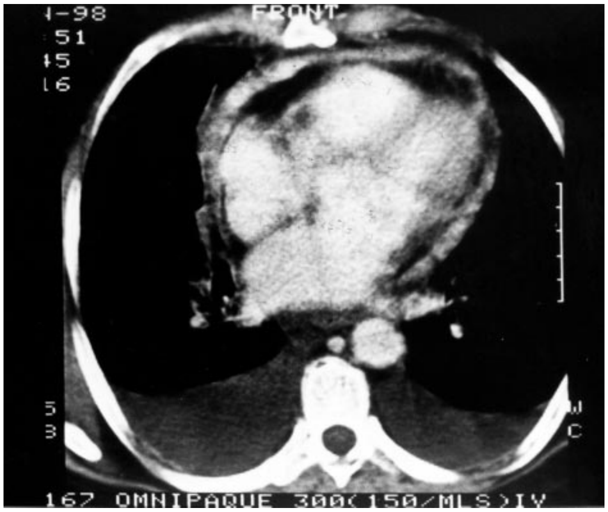

Figure 2. CT scan of heart, showing thickened pericardium.

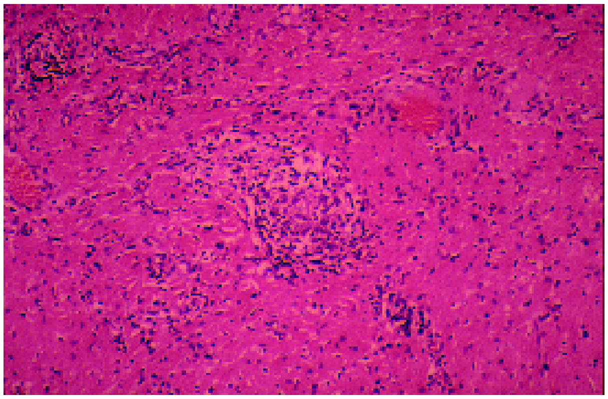

Figure 3. Histologic results of pericardial biopsy, which show granulomatous pericarditis.

### Case 2

A 50-year-old Omani man from a rural area was hospitalized on June 2, 1999, with a 3-week history of fever of unknown cause, not responding to cefuroxime. Hematologic examination revealed hemoglobin 13 g/dl, white blood cells 6.7 x $10^9$/L (neutrophils 53%, lymphocytes 37%, monocytes 9%), platelets 195 x $10^9$/L, and erythrocyte sedimentation rate 2 mm/hour. Signs of bilateral basal pneumonia developed, and Q-fever serologic tests were positive: total antibody titers to phase-II antigen by IF ≥ 1:1280 (threshold ≥ 1:80), and IgM titer ≥ 1:320 (threshold ≥ 1:20). After treatment with doxycycline, the patient's symptoms resolved. Repeated serologic results were total IF antibody titer 1:2560 and IgM titer 1:160, evidence of the convalescent phase of infection.

### Conclusions

In most countries on the Arabian Peninsula, including Oman, most people live in rural areas and are exposed to livestock. In countries where it is endemic, Q fever usually occurs sporadically and may be underdiagnosed and underreported (6,8). In Zimbabwe, where acute Q fever had never been reported, a recent community survey revealed *C. burnetii* antibodies in 37% of humans, 39% of cattle, and 10% of goats (12).

In Case 1, pericardial involvement in the acute stage of Q fever likely progressed to chronic constrictive pericarditis because diagnosis and appropriate treatment were delayed. For acute Q fever, the treatment of choice is doxycycline or orfloxacin, alone or in combination with rifampicin, for 21 days. For treatment of chronic Q fever (e.g., with endocarditis), doxycycline and a quinolone for a minimum of 3 months have been advocated (5-7).

Q fever may be widely endemic but undiagnosed in livestock and domestic animals in Oman. Similarly, the evidence suggests that acute Q fever is prevalent, but undiagnosed (or unreported) in other Arabian Peninsula countries, including Saudi Arabia. Diagnostic serologic tests are not yet available in Oman but will be introduced soon, which will permit us to determine prevalence and distribution of *C. burnetii* in humans and in livestock.

### Acknowledgments

The authors thank Didier Raoult, Centre for Rickettsial Studies, University of Marseilles, for PCR testing of the biopsy specimen. The Marcel Merieux Laboratory, Lyon, France, conducted the immunofluorescence tests, and Elizabeth Richens provided sera from Case 1 for immunologic studies.

Dr. Scrimgeour is an associate professor in Infectious and Tropical Diseases, and heads the Communicable Diseases unit at the Sultan Qaboos University. His research interests include epidemiology of infectious and tropical diseases and parasitic disease of the central nervous system.

### References

1. Lippe M, Seebastiani A, El-Mutabakani H. Investigation of serum antibodies to rheovirus, adenovirus, and *Coxiella burnetii* in a group of inhabitants of Riyadh, Saudi Arabia. Arch Ital Sc Med Trop 1968;49:129-36.
2. Gelpi AP. Q fever in Saudi Arabia. Am J Trop Med Hyg 1966;15:784-98.
3. Greth A, Calvez D, Vassart M, Lefevre PC. Serological survey for bovine bacterial pathogens in captive Arabian oryx (*Oryx leuocoryx* Pallas, 1776). Revue Scientifique et Technique Office International des Epizooties (France). 1992:11:1163-8.
4. Afzal M, Sakkir M. Survey of antibodies to various infectious disease agents in racing camels in Abu Dhabi, UAE. Rev Sci Tech 1994;13:787-92.
5. Dupuis G, Peter O, Pedroni D. Aspects cliniques observes lors d'une epidemie de 415 cas de fievre Q. Schweiz Med Wschr 1985;115:814-8.
6. Marrie TJ. *Coxiella burnetii* (Q fever). In: Mandell GL, Bennett JE, Dolin R, editors). Principles and practice of infectious diseases. New York: Churchill Livingstone Inc.; 1995. p. 1727-35.
7. Pinto JR. Pleuropericardial lesions in Q fever. BMJ 1973;71;2:1542.
8. Brouqui P, Tissot-Dupont H, Drancourt M, Berland Y, Etienne J, Leport C, et al. Chronic Q fever: ninety-two cases from France including 27 cases without endocarditis. Arch Intern Med 1993;153:642-8.
9. Diaz Morant V, Mateo Sanchez JI, Lara Fernandez A, Cabello Rueda F. Pleuropericarditis caused by Q fever [in Spanish] An Med Interna 1995;12:568-9.
10. Beamon MH, Hung J. Pericarditis associated with tick-borne Q fever. Aus NZ J Med 1989;19:254-6.
11. Tissot-Dupont H, Thirion X, Raoult D. Q fever serology cut-off determination for microimmunofluorescence. Clin Diagn Lab Immunol 1994;1:189-96.
12. Kelly PJ, Matthewman LA, Mason PR, Raoult D. Q fever in Zimbabwe. S Afr Med J 1993;83:21-5.
